# Changes in 3-month mineral and bone disorder patterns were associated with all-cause mortality in prevalent hemodialysis patients with secondary hyperparathyroidism

**DOI:** 10.1186/s12882-020-02088-x

**Published:** 2020-10-12

**Authors:** Chihiro Kato, Naohiko Fujii, Chisato Miyakoshi, Shinji Asada, Yoshihiro Onishi, Shingo Fukuma, Takanobu Nomura, Michihito Wada, Masafumi Fukagawa, Shunichi Fukuhara, Tadao Akizawa

**Affiliations:** 1 Medical Affairs Department, Kyowa Kirin Co., Ltd, Otemachi Financial City Grand Cube, 1-9-2 Otemachi, Chiyoda-ku, Tokyo, 100-0004 Japan; 2 Department of Internal Medicine (Nephrology Unit), Hyogo Prefectural Nishinomiya Hospital, Nishinomiya, Japan; 3 Department of Healthcare Epidemiology, School of Public Health, Faculty of Medicine, Kyoto University, Kyoto, Japan; 4 Department of Pediatrics, Kobe City Medical Center General Hospital, Kobe, Japan; 5 Institute for Health Outcomes and Process Evaluation Research (iHope International), Kyoto, Japan; 6 Human Health Sciences, Kyoto University Graduate School of Medicine, Kyoto, Japan; 7 The Keihanshin Consortium for Fostering the Next Generation of Global Leaders in Research (K-CONNEX), Kyoto, Japan; 8 Division of Nephrology, Endocrinology and Metabolism, Tokai University School of Medicine, Isehara, Japan; 9 Division of Nephrology, Department of Medicine, Showa University School of Medicine, Tokyo, Japan

**Keywords:** Changing pattern, CKD-MBD, Hemodialysis, Secondary hyperparathyroidism, Mortality

## Abstract

**Background:**

There is limited evidence on the association between short-term changes in mineral and bone disorder parameters and survival in maintenance hemodialysis patients.

**Methods:**

We investigated the association between changing patterns of phosphorus, calcium and intact parathyroid hormone levels and all-cause mortality in hemodialysis patients with secondary hyperparathyroidism. Each parameter was divided into three categories (low [L], middle [M] and high [H]), and the changing patterns between two consecutive visits at 3-month intervals were categorized into nine groups (e.g., L-L and M-H). The middle category was defined as 4.0–7.0 mg/dL for phosphorous, 8.5–9.5 mg/dL for calcium and 200–500 pg/mL for intact parathyroid hormone. Adjusted incidence rates and rate ratios were analyzed by weighted Poisson regression models accounting for time-dependent exposures.

**Results:**

For phosphorus, shifts from low/high to middle category (L-M/H-M) were associated with a lower mortality compared with the L-L and H–H groups, whereas shifts from middle to low/high category (M-L/M-H) were associated with a higher mortality compared with the M-M group. For calcium, shifts from low/middle to high category (L–H/M-H) were associated with a higher mortality compared with the L-L and M-M groups, whereas shifts from high to middle category (H-M) were associated with a lower mortality compared with the H–H group. For intact parathyroid hormone, shifts from low to middle category (L-M) were associated with a lower mortality compared with the L-L group.

**Conclusions:**

Changes in the 3-month patterns of phosphorus and calcium toward the middle category were associated with lower mortality. Our study also suggests the importance of avoiding hypercalcemia.

## Background

High serum phosphorus (P) and calcium (Ca) concentrations measured at single time points are associated with all-cause and cardiovascular mortality in hemodialysis patients [1, 2]. It has been shown that the importance of maintaining P and Ca levels on prognosis by measuring their concentrations at multiple time points [3–6].

In the Mineral and Bone Disorder Outcomes Study for Japanese CKD Stage 5D Patients (MBD-5D), a prospective observational study conducted in hemodialysis patients with secondary hyperparathyroidism (SHPT) in Japan, high P and Ca levels correlated with increased mortality, while no clear correlation was observed for high intact parathyroid hormone (iPTH) levels [3]. Moreover, it has been found that instability in P, Ca and iPTH levels can influence prognosis, and the importance of maintaining P and Ca levels within specific ranges has been consistently suggested in the prospective observational studies conducted in Europe and North America [7–10] For iPTH, the only observation was that low iPTH levels may pose a risk of increased mortality [10]. However, in these prospective observational studies, variations in P, Ca and iPTH levels were evaluated at 6- or 12-month intervals, which were longer than in daily clinical practice [7, 8, 10].

The Kidney Disease Improving Global Outcomes (KDIGO) Clinical Practice Guidelines for chronic kidney disease–mineral and bone disorder (CKD-MBD) suggested that the reasonable frequency for evaluating these parameters is every 1–3 months for Ca and P and every 3–6 months for PTH, although the evidence level was “Not Graded” [11]. Therefore, we investigated the association between all-cause mortality and changes in MBD-related parameters every 3 months, which is more frequent than the previously-reported 6- or 12-month intervals, in Japanese hemodialysis patients with SHPT.

## Methods

### Study design and study population

MBD-5D was a multicenter, prospective observational study of maintenance hemodialysis patients with SHPT [12]. The eligibility criteria were as follows: (i) patients undergoing hemodialysis and (ii) patients with iPTH ≥ 180 pg/mL (according to the Japanese Society for Dialysis Therapy [JSDT] guidelines [13]) or receiving vitamin D receptor activator (VDRA) treatment. A total of 8229 patients from 86 facilities were registered, and their clinical outcomes, including all-cause mortality, were collected for 3 years. Data on prescribed medication and MBD-related parameters (Ca, P and iPTH) were collected every 3 months; data on other time dependent variables were collected every 6 months. Patient characteristics were shown in Table 1.

**Table 1:** Baseline characteristics of the study population

Variable	Value
Demographics	
Age, years	63 (55–71)
Sex, female, %	37.7
Body mass index, kg/m^2^	20.9 (19.0–23.3)
Cause of end-stage kidney disease, %	
Glomerulonephritis	45.1
Diabetic nephropathy	24.2
Other disease	30.7
Comorbidities, %	
Diabetes mellitus	31.6
Cardiovascular disease	60.0
Lung disease	7.4
Liver disease	14.1
Malignancy	5.1
Dialysis	
Duration of dialysis, years	8.3 (3.7–14.3)
Kt/V, single pool	1.4 (1.2–1.6)
Dialysate Ca, %	
< 3.0 mEq/L	52.0
≥ 3.0 mEq/L	48.0
Laboratory data	
Serum albumin, g/dL	3.8 (3.5–4.0)
Blood hemoglobin, g/dL	10.5 (9.8–11.2)
MBD-related characteristics	
Serum parameters	
Ca, mg/dL^a^	9.4 (8.9–10.1)
P, mg/dL	5.5 (4.6–6.3)
iPTH, pg/mL	267 (197–401)
History of parathyroidectomy, %	6.2
Prescription of MBD-related agents, %	
VDRA	77.6
Intravenous	48.4
Oral	30.4
Phosphate binder	85.1
Ca carbonate	66.7
Sevelamer hydrochloride	41.1
Cinacalcet hydrochloride^b^	0.0

Data were derived from the subcohort (*n* = 3276) randomly selected from the study population

Continuous variables were expressed as median (interquartile range)

*iPTH* Intact parathyroid hormone, *Ca* Calcium, *MBD* Mineral and bone disorder, *P* Phosphorus, *VDRA* Vitamin D receptor activator

^a^Albumin-corrected value; calcium + (4.0—albumin), if albumin is less than 4.0 g/dL

^b^Became available in January 2008 in Japan

**Fig. 1:**
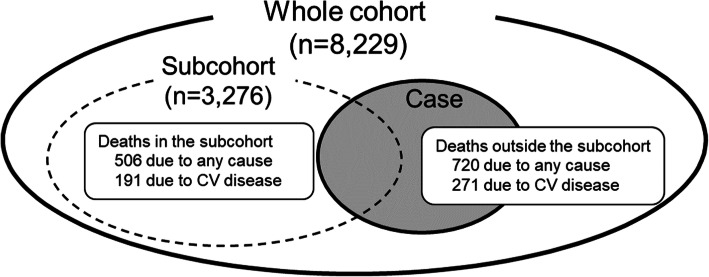
Study design of the MBD-5D study.The study has a whole cohort (solid circle) of all patients enrolled and a subcohort (dotted circle) of randomly selected 40% of the whole cohort. From 86 facilities, all 8,229 dialysis patients with secondary hyperparathyroidism were registered and 3,276 patients were selected into the subcohort. In total, 1,226 all-cause deaths and 462 deaths due to cardiovascular disease were reported. CV, cardiovascular

This study was designed as a case-cohort study; 40% of the whole cohort (*n* = 3276) was randomly selected into a subcohort, from which data were collected prospectively (Fig. 1). Data from patients who died and patients outside the subcohort were collected retrospectively.

### Exposures, outcomes and covariates

**Fig. 2:**
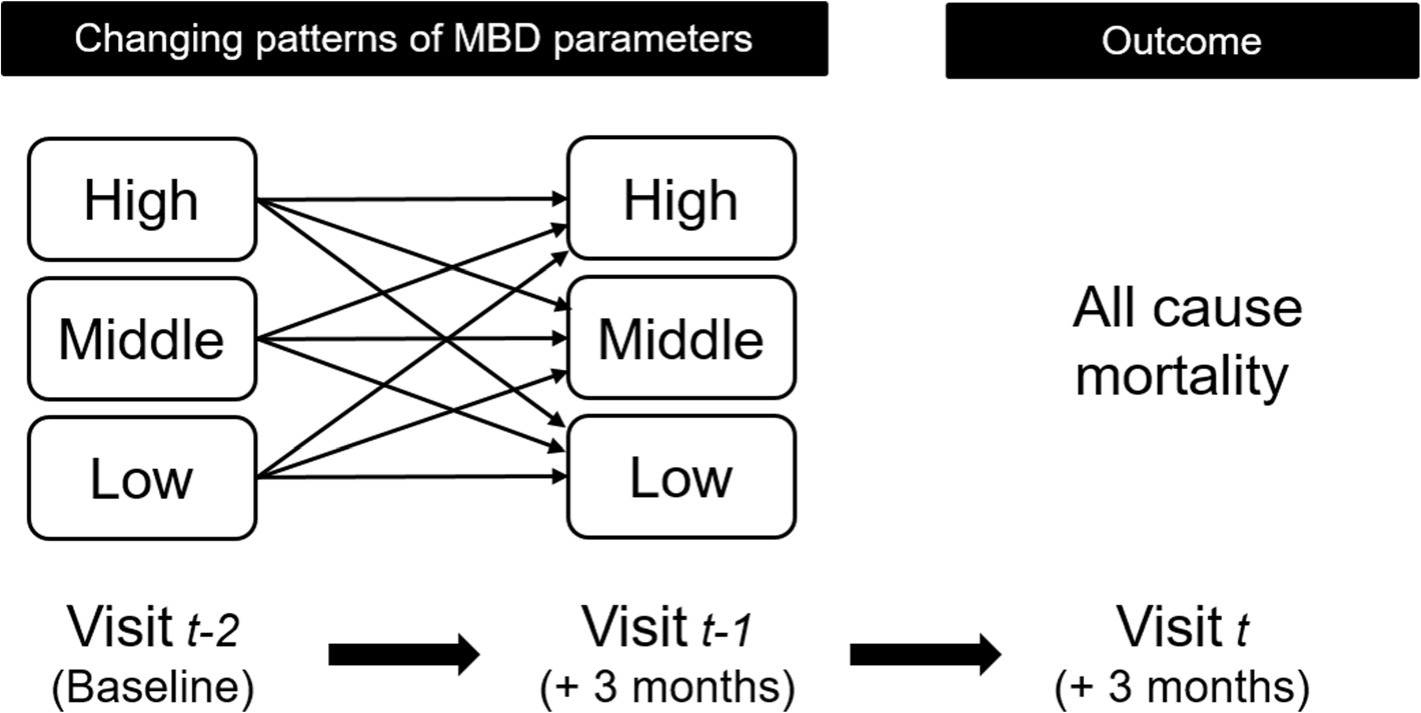
Illustration of changing patterns of MBD parameters and outcome. Levels of each parameter were divided into three categories (Low, Middle and High); the middle categories were defined as 4.0–7.0 mg/dL for phosphorus, 8.5–9.5 mg/dL for calcium and 200–500 pg/mL for intact parathyroid hormone. MBD, mineral and bone disorder

The outcome of interest was all-cause mortality. MBD-related parameters were considered time-dependent variables. Levels of each parameter were divided into three categories: low (L), middle (M) and high (H), so that the middle categories (4.0–7.0 mg/dL for P, 8.5–9.5 mg/dL for Ca [14] and 200–500 pg/mL for iPTH) were compatible with the middle category based on positive stratification for mortality in the previous report [6]. The changing patterns of each parameter were categorized into nine classes according to the level of that parameter measured at two consecutive visits [3]. For example, if the level of a parameter was “L” at one visit and “H” at the following visit, then the pattern would be categorized as “L–H” (Fig. 2).

Covariates included fixed patient characteristics (age, sex, primary kidney disease, diabetes, dialysis vintage, cardiovascular disease, lung disease, liver disease, malignancy and history of parathyroidectomy) and time-dependent variables that were updated at each visit (VDRAs, phosphate binders, cinacalcet, albumin, hemoglobin, body mass index, Kt/V and dialysate Ca concentration).

### Statistical analyses

To estimate the effect of MBD parameters on mortality, time-dependent Poisson regression models were used for time-dependent exposure and covariates such as MBD treatment by weighted inverse probability of having the specific pattern of change observed for each MBD parameter in the study.

For each 3-month interval ending at visit *t*, the incidence of clinical outcome was modeled based on the changing patterns of MBD parameters during the previous 3-month interval from visit *t—2* (baseline) to visit *t—1*. The probability of having the pattern observed in each patient was calculated using pooled multinomial logistic regression models; for each MBD parameter, the other MBD parameter levels and covariates mentioned in the previous section at visit t—2 were used as independent variables in these models.

Weighted Poisson regression was used to estimate adjusted incidence rates (aIRs) and adjusted incidence rate ratios (aIRRs). The weights were calculated using the inverse probability of having the specific pattern for each MBD parameter and the inverse of the sampling fraction (1 / 0.4 = 2.5) for controls in the subcohort. To handle within-patient correlation, generalized estimating equations with independent working correlation and robust variance estimator were used. The results of regression analyses were presented as point estimates and 95% confidence intervals (95% CIs).

A post hoc analysis was performed to explore factors associated with higher Ca levels (greater than the middle category). Background characteristics of the patients in the M-H group were compared with those in the M-L/M-M groups by calculating the standard mean difference (SMD). An SMD > 0.2 might suggest non-trivial effect size [15].

A sensitivity analysis was conducted to assess the robustness of results with longer intervals; the association between the changing patterns of MBD parameters observed during a 12-month interval and the incidence of clinical outcomes during the next 12-month interval was examined by using similar models as used for the main analysis. SAS version 9.4 (SAS Institute Inc) was used for all analyses.

## Results

### Baseline characteristics and study outcome

Median P, Ca and iPTH levels were 5.5 mg/dL, 9.4 mg/dL and 267 pg/mL, respectively, at the time of enrollment (Table 1). At the last visit, median P and Ca levels remained nearly the same (5.2 mg/dL and 9.5 mg/dL, respectively), while median iPTH decreased to 165 pg/mL.

During the 3-year study period, a total of 1226 all-cause deaths were observed (506 among patients in the subcohort and 720 among those outside the subcohort). Total observation period was 20 444 person-years. The overall mortality rate was 5.5 events/100 person-years.

### Association between mortality and changing patterns of MBD parameters

#### Serum phosphorus

The aIR for patients whose P levels were maintained in the middle (M-M) category during the 3-month intervals was 4.9/100 person-years, while the aIRs for those whose P levels were maintained in the low (L-L) and high (H–H) categories were 7.4 and 11.1/100 person-years, respectively (Table 2). The risk of mortality was significantly higher in the L-L and H–H groups than in the M-M group (aIRR [95% CI]: 1.53 [1.14–2.03] and 2.28 [1.49–3.49], respectively).

**Table 2:** aIRs of mortality for patients whose parameters were maintained in the same category during the 3-month intervals

	Adjusted incidence rates [95% CI], /100 person-years		
	L-L group	M-M group	H–H group
P	7.4 [5.4–9.4]	4.9 [4.4–5.3]	11.1 [6.4–15.7]
Ca	3.7 [1.5–5.8]	4.5 [3.9–5.2]	6.8 [6.1–7.5]
iPTH	5.9 [5.3–6.5]	5.4 [4.7–6.1]	6.7 [4.1–9.3]

Levels of each parameter were divided into three categories (L, M and H); the middle category were defined as 4.0–7.0 mg/dL for P, 8.5–9.5 mg/dL for Ca and 200–500 pg/mL for iPTH. For each 3-month interval ending at visit *t*, the changing patterns of MBD parameters during the previous 3-month interval from visit *t—2* (baseline) to visit *t—1* (3 months later) were evaluated. Incidence rates were adjusted for patients’ characteristics (age, sex, primary kidney disease, diabetes, dialysis vintage, cardiovascular disease, lung disease, liver disease, malignancy and history of parathyroidectomy) and time-varying variables (VDRAs, phosphate binders, cinacalcet, albumin, hemoglobin, body mass index, Kt/V and dialysate Ca concentration)

*aIRs* Adjusted incident rates, *Ca* Calcium, *H* High, *iPTH* Intact parathyroid hormone, *L* low, *M* Middle, *MBD* Mineral and bone disorder, *P* Phosphorus, *VDRA* Vitamin D receptor activator

**Fig. 3:**
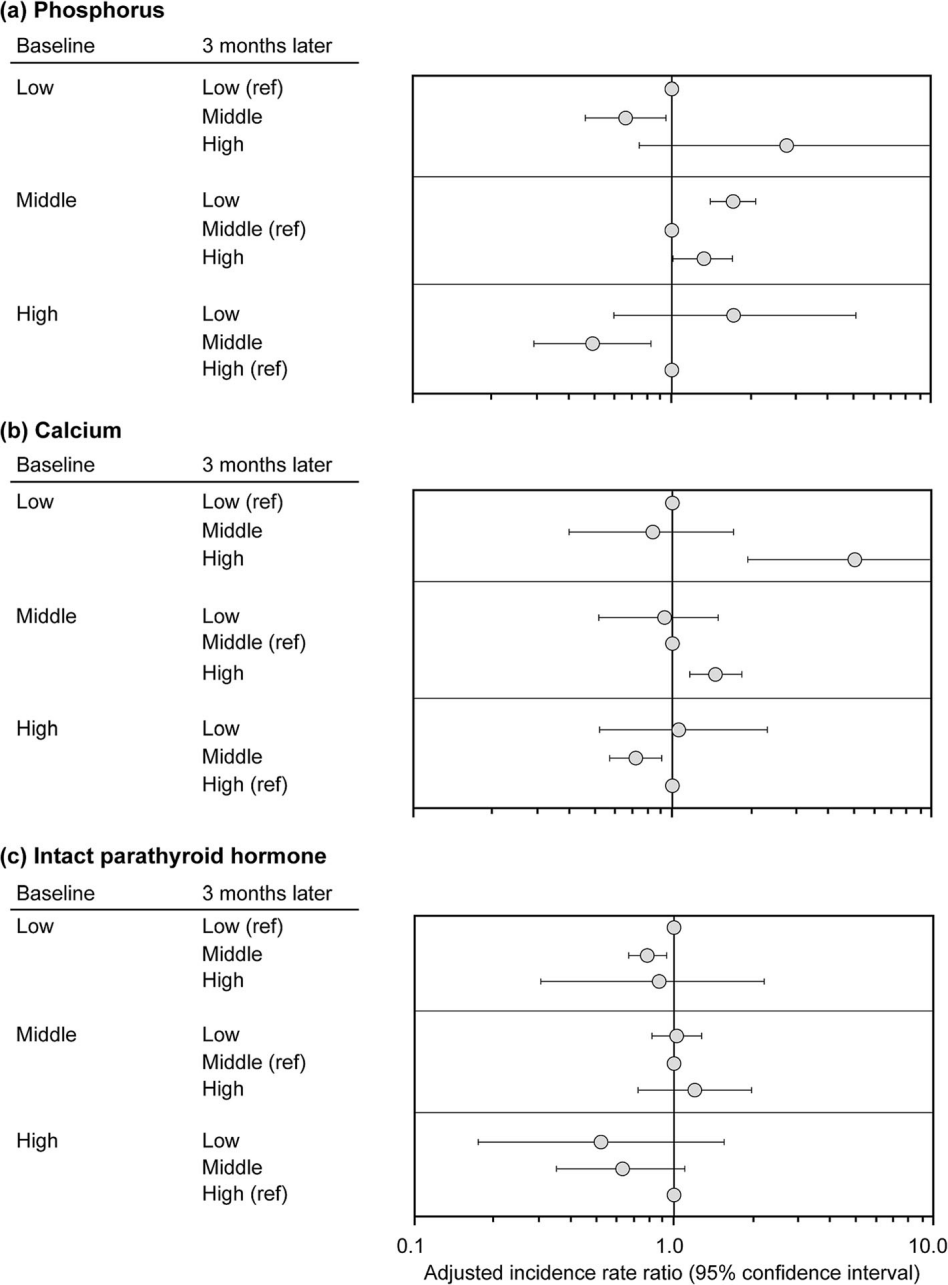
aIRRs for each combination of the values at baseline and 3 months later. Levels of each parameter were divided into three categories (L, M and H); the middle category were defined as 4.0–7.0 mg/dL for phosphorus, 8.5–9.5 mg/dL for calcium and 200–500 pg/mL for intact PTH. For each 3-month interval ending at visit *t*, the changing patterns of MBD parameters during the previous 3-month interval from visit *t—2* (baseline) to visit *t—1* (3 months later) were evaluated. Incidence rate ratios were adjusted for patients’ characteristics (age, sex, primary kidney disease, diabetes, dialysis vintage, cardiovascular disease, lung disease, liver disease, malignancy and history of parathyroidectomy) and time-varying variables (VDRAs, phosphate binders, cinacalcet, albumin, hemoglobin, body mass index, Kt/V and dialysate Ca concentration). aIRRs, adjusted incident rate ratios; H, high; PTH, parathyroid hormone; L, low; M, middle; MBD, mineral and bone disorder; ref, reference; VDRA, vitamin D receptor activator

Patients with P levels shifting from the middle to low/high (M-L/M-H) category had a higher risk of mortality compared with those in the M-M group (Fig. 3a, middle). Changes in patterns from the low/high to middle category (L-M/H-M) were significantly associated with a lower risk of mortality compared with the unchanged patterns (L-L/H–H) (Fig. 3a, top/bottom).

#### Serum calcium

The aIRs for patients whose Ca levels were maintained in the same category during the 3-month intervals were 3.7/100 person-years for the low category, 4.5/100 person-years for the middle category and 6.8/100 person-years for the high category (Table 2). The mortality risk increased linearly according to Ca levels. There was no significant difference between the L-L and M-M groups. On the other hand, the aIR in the H–H group was significantly higher than that in the M-M group (aIRR [95% CI]: 1.51 [1.27–1.79]).

Among patients with Ca levels in the middle category at baseline, those who shifted to the high (M-H) category 3 months later were at a significantly higher risk compared with those who remained in the M-M group (Fig. 3b, middle). Among patients with Ca levels in the low category at baseline, those who shifted to the high category had a higher risk of mortality compared with those who remained in the low (L-L) category; however, there was no significant difference between the L-L and L-M groups (Fig. 3b, top). Among patients with Ca levels in the high category at baseline, those who shifted to the middle (H-M) category had a lower risk of mortality compared with those who remained in the high (H–H) category (Fig. 3b, bottom).

Table 3 shows a comparison of the background characteristics of patients in the M-H group versus the M-L/M-M groups. The former group was administered intravenous VDRA and cinacalcet more often than the latter groups (SMD: 0.22 and 0.21, respectively).

**Table 3:** Characteristics of patients shifted toward to high category in Ca level during the 3-month intervals

	M-L and M-M groups	M-H group	SMD
Demographics			
Age, years	62.0 ± 20.2	60.8 ± 19.6	0.06
Sex, female, %	36.5	40.4	0.15
Body mass index, kg/m^2^	21.5 ± 5.7	21.4 ± 5.6	0.01
Cause of end-stage kidney disease, %			
Glomerulonephritis	39.3	46.6	
Diabetic nephropathy	29.4	22.6	0.17
Others	31.4	30.8	
Comorbidities, %			
Diabetes mellitus	36.9	28.5	0.18
Cardiovascular diseases	58.6	57.8	0.02
Lung diseases	7.2	7.0	0.01
Liver diseases	12.8	13.8	0.03
Malignancies	4.7	4.1	0.03
Dialysis			
Log duration of dialysis, years	0.7 ± 0.7	0.7 ± 12.6	0.14
Kt/V, single pool	1.4 ± 0.5	1.5 ± 0.5	0.07
Dialysate calcium, mEq/L	2.7 ± 0.4	2.8 ± 0.4	0.03
Laboratory data			
Albumin, g/dL	3.7 ± 0.5	3.7 ± 0.5	0.00
Hemoglobin, g/dL	10.5 ± 1.8	10.6 ± 1.8	0.05
MBD-related characteristics			
Serum parameters			
P, mg/dL	5.4 ± 2.2	5.6 ± 2.2	0.09
Log iPTH, pg/mL	2.3 ± 0.5	2.3 ± 0.5	0.05
History of parathyroidectomy, %	4.4	6.2	0.08
Prescription of MBD-related agents, %			
VDRAs	79.8	86.4	0.22
Intravenous	45.3	55.4	
Oral	34.5	31.0	
Phosphate binders	85.8	89.5	0.16
Both	26.4	31.4	
Non–Ca-based	15.0	17.3	
Ca-based	44.4	40.7	
Cinacalcet	19.3	28.2	0.21

Values are presented as mean ± standard deviation for continuous variables and proportions for categorical variables

*Ca* Calcium, *H* High, *iPTH* Intact parathyroid hormone, *L* Low, *M* Middle, *MBD* Mineral and bone disorder, *P* Phosphorus, *SMD* Standard mean difference, *VDRA* Vitamin D receptor activator

#### Serum intact parathyroid hormone

The aIRs for patients whose iPTH levels were maintained in the same category during the 3-month intervals were 5.9/100 person-years for the low category, 5.4/100 person-years for the middle category and 6.7/100 person-years for the high category (Table 2). Compared with patients whose iPTH levels were maintained in the middle (M-M) category, those whose iPTH levels were maintained in the low (L-L) and high (H–H) categories did not show any significant differences in mortality risk (aIRR [95% CI]: 1.09 [0.93–1.27] and 1.24 [0.82–1.86], respectively).

Among patients with iPTH levels in the middle category at baseline, those who shifted to the low (M-L) or high (M-H) category did not show any difference in the risk of mortality compared with the M-M group (Fig. 3c, middle). However, among patients with iPTH levels in the low category at baseline, those who shifted to the middle (L-M) category had a significantly lower risk of mortality compared with those who remained in the low (L-L) category (Fig. 3c, top). Among patients with iPTH levels in the high category at baseline, those who shifted to the middle (H-M) or low (H–L) category had a lower, albeit statistically insignificant, risk of mortality compared with those who remained in the high (H–H) category (Fig. 3c, bottom).

### Sensitivity analysis with 12-month intervals

#### Serum phosphorus

**Fig. 4:**
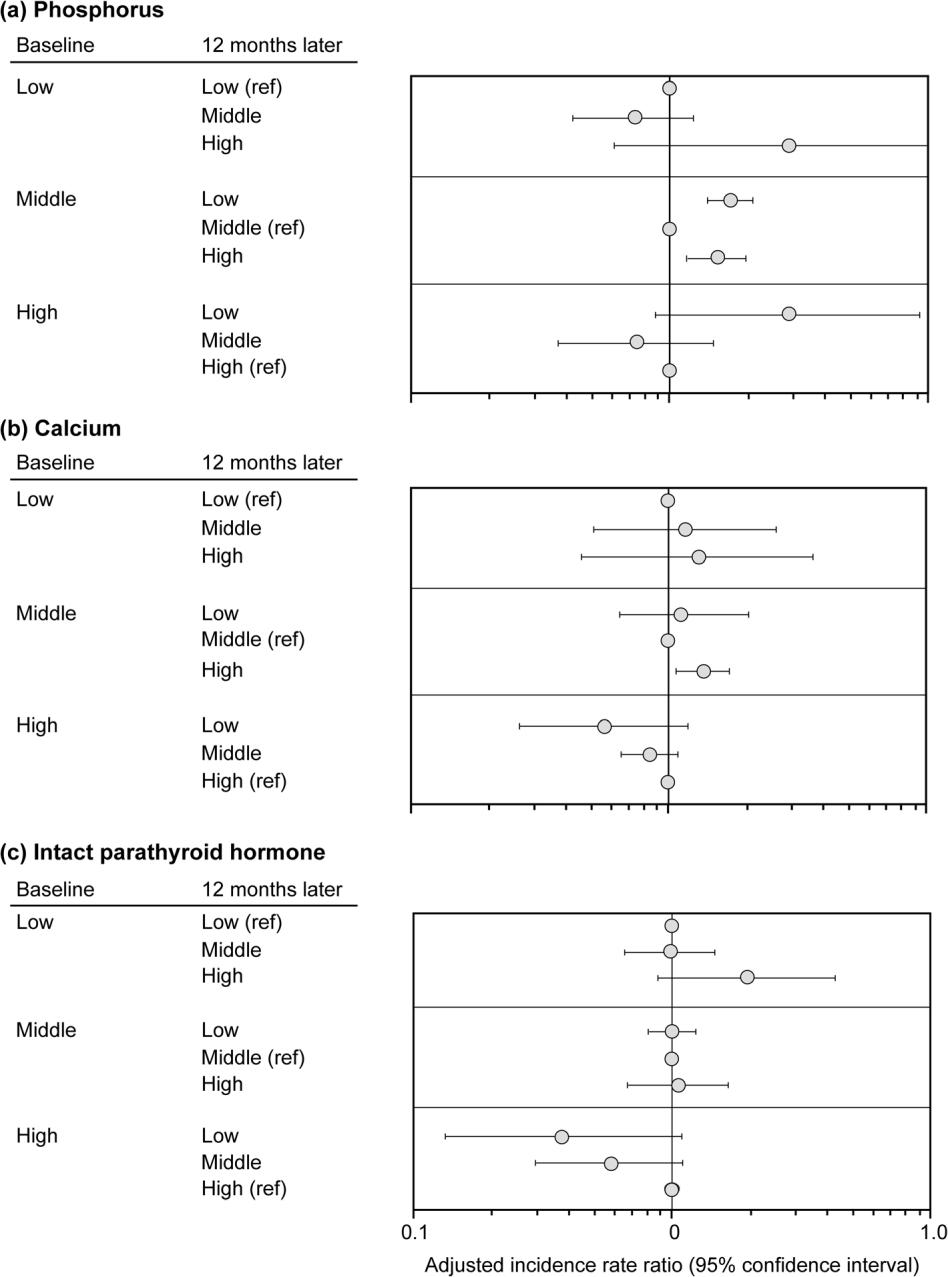
aIRRs for each combination of the values at baseline and 12 months later. Levels of each parameter were divided into three categories (L, M and H); the middle category were defined as 4.0–7.0 mg/dL for phosphorus, 8.5–9.5 mg/dL for calcium and 200–500 pg/mL for intact PTH. For each 12-month interval ending at visit *t*, the changing patterns of MBD parameters during the previous 12-month interval from visit *t—2* (baseline) to visit *t—1* (12 months later) were evaluated. Incidence rate ratios were adjusted for patients’ characteristics (age, sex, primary kidney disease, diabetes, dialysis vintage, cardiovascular disease, lung disease, liver disease, malignancy and history of parathyroidectomy) and time-varying variables (VDRAs, phosphate binders, cinacalcet, albumin, hemoglobin, body mass index, Kt/V and dialysate Ca concentration). aIRRs, adjusted incident rate ratios; H, high; PTH, parathyroid hormone; L, low; M, middle; MBD, mineral and bone disorder; ref, reference; VDRA, vitamin D receptor activator

In the sensitivity analysis with 12-month intervals, the trend of aIRs among patients whose P levels were maintained in the same category was similar to that of the analysis with 3-month intervals (Table 4). However, the aIRR for the H–H group was attenuated and was not significantly different (aIRR [95% CI]: 1.81 [0.95–3.42]), and the significant differences observed between the L-M and L-L/H-M groups and between the H-M and H–H groups in the analysis with 3-month intervals were not observed in the sensitivity analysis (Fig. 4a, top/bottom).

**Table 4:** aIRs of mortality for patients whose parameters were maintained in the same category during the 12-month intervals

	Adjusted incidence rates [95% CI], /100 person-years		
	L-L group	M-M group	H–H group
P	8.7 [4.7–12.7]	5.6 [5.1–6.1]	10.1 [3.7–16.5]
Ca	7.6 [3.1–12.2]	5.3 [4.4–6.3]	6.7 [5.9–7.5]
iPTH	6.2 [5.3–7.1]	6.5 [5.5–7.4]	13.1 [6.7–19.6]

Levels of each parameter were divided into three categories (L, M and H); the middle category were defined as 4.0–7.0 mg/dL for P, 8.5–9.5 mg/dL for Ca and 200–500 pg/mL for iPTH. For each 12-month interval ending at visit *t*, the changing patterns of MBD parameters during the previous 12-month interval from visit *t—2* (baseline) to visit *t—1* (12 months later) were evaluated. Incidence rates were adjusted for patients’ characteristics (age, sex, primary kidney disease, diabetes, dialysis vintage, cardiovascular disease, lung disease, liver disease, malignancy and history of parathyroidectomy) and time-varying variables (VDRAs, phosphate binders, cinacalcet, albumin, hemoglobin, body mass index, Kt/V and dialysate Ca concentration)

*aIRs* Adjusted incident rates, *Ca* Calcium, *H* High, *iPTH* Intact parathyroid hormone, *L* Low, *M* Middle, *MBD* Mineral and bone disorder, *P* Phosphorus; VDRA, vitamin D receptor activator

#### Serum Calcium

In the sensitivity analysis with 12-month intervals, the H–H group had a significantly higher risk of mortality compared with the M-M group (aIRR [95% CI]: 1.25 [1.00–1.56] for the H–H group); however, the magnitude of difference was smaller than in the analysis with 3-month intervals (Table 4). There were no significant differences in mortality between the L–H and L-L groups and between the H-M and H–H groups in the sensitivity analysis (Fig. 4b, top/bottom).

#### Serum intact parathyroid hormone

In the sensitivity analysis with 12-month intervals, the H–H group had a significantly higher risk of mortality compared with the M-M group (aIRR [95% CI]: 2.03 [1.22–3.39]) (Table 4). There was no significant difference in mortality between the L-M and L-L groups in the sensitivity analysis (Fig. 4c, top).

## Discussion

In the present study, we observed a significant association between all-cause mortality and changes in the 3-month patterns of P and Ca in Japanese hemodialysis patients with SHPT. Our results show that evaluating the patterns of MBD-related parameters at shorter intervals can show significant association with mortality than at longer intervals, such as 6 or 12 months.

In the present study, we found consistent, bidirectional and significant associations between the changing patterns of serum P and all-cause mortality. Briefly, the patients who shifted from the middle category (M-H and M-L groups) had a higher mortality than those who remained in the middle category (M-M group), and the patients who shifted to the middle category (H-M and L-M groups) had a lower mortality than those who persisted in the original category (H–H or L-L group). Although these results clearly demonstrated the clinical relevance of maintaining P levels in the middle category, it is unclear whether the middle category (4.0–7.0 mg/dL) used in the present study is optimal for other dialysis patients, as it is higher than the target ranges set in the JSDT (3.5–6.0 mg/dL) and Kidney Disease Outcomes Quality Initiative (KDOQI) (3.5 5.5 mg/dL) guidelines and the safety zone in the COSMOS (3.6–5.2 mg/dL) study [7]. However, the optimal (middle) range in the present study was chosen according to the range of lower mortality risk in the previous study analyzed MBD-5D [6]. One of the reason of the difference of P range between our study and clinical guidelines is that the study population of MBD-5D was limited to dialysis patients with SHPT who are at a higher risk of cardiovascular disease [16] and have the much severe condition of the serum *P* value [6].

Higher serum Ca levels were consistently associated with higher all-cause mortality; the L–H group had a higher risk than the L-L group, the M-H group had a higher risk than the M-M group and the H-M group had a lower risk than the H–H group. These results were in line with previous reports by JSDT, DOPPS and MBD-5D, suggesting that even in a short observation period of 3 months, high Ca levels are consistently associated with higher mortality, and optimization of Ca levels is important for lower mortality. The middle category used for Ca in this study (8.5–9.5 mg/dL) was similar to the target range set in KDOQI (8.4-9.5 mg/dL). The upper limit of the middle category for Ca in this study was lower than that of the target range set for Ca in the 2006 JSDT guidelines (8.4–10.0 mg/dL) [13]. However, the revised JSDT guidelines recommend that Ca levels in dialysis patients should be maintained at the lowest possible within the target range [17]. In this study, the mortality risk in the H–H and M-H group was significantly higher than that in the M-M group. In Europe, the safety zone for Ca was indicated at 7.9 9.5 mg/dL in maintenance dialysis patients [7]. In the USA, mortality risk in incident hemodialysis patients whose Ca levels were maintained at 9.5–10.2 mg/dL was significantly higher than that in patients whose Ca levels were maintained at 8.4–9.5 mg/dL [9]. Therefore, adequate caution should be exercised if Ca levels exceed 9.5 mg/dL. We explored possible reasons for high Ca levels and found that patients in the M-H group tended to use intravenous VDRA and cinacalcet more often (SMD > 0.2). These results supported the importance of avoiding hypercalcemia as recommended by the 2017 KDIGO guidelines [11].

For iPTH, no differences were observed in mortality risk between the L-L, M-M and H–H groups. These results are not consistent with reports from Europe and North America, where decreased iPTH levels correlated with higher cardiovascular risk and all-cause mortality [8, 10]. One possible reason is that the target population in the MBD-5D study was limited to Japanese hemodialysis patients. In Japan, iPTH levels were maintained at a lower range compared with those in Western countries [18], and achievement of the iPTH target range led to low mortality [5]. Moreover, 42% of the patients had started cinacalcet during the study period [19]. In these patients, the MBD parameters, including PTH, Ca and P levels, reduced after cinacalcet administration [20]. Since the changing patterns of each MBD parameter were analyzed independently of other MBD parameters, the benefits of controlling PTH may have been underestimated.

Results of the sensitivity analysis with 12-month intervals were generally similar to those of the 3-month intervals. However, attenuation of aIRRs was observed in some groups, such as L-M and H-M for P levels and L–H and H-M for Ca levels. This could be due to possible misclassification of the MBD parameters during the observation period, as they can easily vary based on lifestyle, bone metabolism and medication.

The study has several strengths. First, MBD-5D was performed as a prospective case cohort study, which enabled both elaborate and repeated data collection with few missing data and powerful outcome measurements with less systematic biases at the same time. Second, participants of this study were restricted to hemodialysis patients with SHPT who were generally at risk of abnormal MBD parameters. Therefore, the results of this study can be applied to patients requiring treatment for CKD-MBD. Third, MBD-5D prospectively collected MBD parameters at 3-month intervals, which enhanced the probability of observing significant variations in MBD parameters associated with mortality that might have been overlooked in previous studies evaluating exposures and outcomes at 6- or 12-month intervals.

The study has several limitations. First, it is not possible to measure unknown confounding factors. Second, there is still a possibility of misclassification of MBD parameters, as they were collected every 3 months only and changes in MBD parameters during each 3-month interval could not be detected. The 2017 KDIGO guidelines recommend that treatment for MBD should be considered based on serial assessment of MBD parameters [11]. On the other hand, evaluating variations at intervals shorter than 3 months might result in increased contamination because bone turnover generally takes place in a 3-month cycle. Third, we analyzed the changes in each MBD parameter independently to simplify the analysis model. On the other hand, the 2017 KDIGO guidelines recommend that MBD parameters should be interpreted together. In clinical practice, it is almost impossible to modify only one of the MBD parameters; therefore, caution is warranted while applying the results of this study to clinical practice. Fourth, iPTH levels in Japanese hemodialysis patients were at a lower range compared with patients in Western countries. Hence, the association between changes in iPTH levels and mortality might not be detectable in a Japanese population. Fifth, pathophysiologically, the short-term changes in MBD parameters might reflect clinical practices in MBD treatment, such as therapeutic inertia in refractory patients, and thus overestimate the difference in mortality. To verify the results of this study, a new clinical study should be conducted excluding such patients. Sixth, we collected data on MBD parameters every 3 months, so these may fluctuated in the period, although we evaluated in ther shortest interval cmparing with the existing suduie [7, 8, 10]. Also, the backgrond causing the observed shifts (e.g., therapy driven, disease progression) are unknown.Finally, it is unknown whether the changes in MBD parameters are due to natural course, medication or other reasons; therefore, it is not well established whether phosphate-lowering therapy has the same effect on mortality as observed in patients with spontaneous decrease in serum P.

## Conclusions

Our study emphasizes the importance of the management of MBD parameters again, especially for serum P which may affect the patients prognosis only with the short term deviation, should be managed in the appropriate range avoiding the risk of hypercalcemia for better prognosis in dialysis patients with SHPT.

## Data Availability

The datasets used and/or analyzed during the current study are available from the corresponding author on reasonable request.

## Abbreviations

aIRRs: Adjusted Incidence Rate Ratios
aIRs: Adjusted Incidence Rates
Ca: Calcium
CIs: Confidence Intervals
CKD-MBD: Chronic Kidney Disease–Mineral and Bone Disorder
H: High
iPTH: Intact Parathyroid Hormone
JSDT: Japanese Society for Dialysis Therapy
KDIGO: Kidney Disease Improving Global Outcomes
KDOQI: Kidney Disease Outcomes Quality Initiative
L: Low
M: Middle
MBD: Mineral and Bone Disorder
MBD-5D: Mineral and Bone Disorder Outcomes Study for Japanese CKD Stage 5D Patients
P: Phosphorus
ref: Reference
SHPT: Secondary Hyperparathyroidism
SMD: Standard Mean Difference
VDRA: Vitamin D Receptor Activator
